# The dynamics of external water conduction in the dryland moss Syntrichia

**DOI:** 10.1093/aobpla/plad025

**Published:** 2023-05-22

**Authors:** Javier Jauregui-Lazo, Marielle Wilson, Brent D Mishler

**Affiliations:** Department of Integrative Biology, and University and Jepson Herbaria, 1001 Valley Life Sciences Building, University of California, Berkeley, CA 94720-2465, USA; Department of Plant Biology, and Genome Center, University of California, Davis, CA 95618, USA; Department of Integrative Biology, and University and Jepson Herbaria, 1001 Valley Life Sciences Building, University of California, Berkeley, CA 94720-2465, USA; Department of Botany, University of British Columbia, Vancouver, BC, Canada; Department of Integrative Biology, and University and Jepson Herbaria, 1001 Valley Life Sciences Building, University of California, Berkeley, CA 94720-2465, USA

**Keywords:** Capillary system, dehydration, ectohydry, hydration, Syntrichia

## Abstract

*Syntrichia* relies on external water conduction for photosynthesis, survival, and reproduction, a condition referred to as ectohydry. Capillarity spaces are abundant in *Syntrichia*, but the link between function and morphology is complex. The aim of this study was to provide a better understanding of species-specific morphological traits underlying the functions of water conduction and storage. We used an environmental scanning electron microscope and confocal microscopy for observing anatomical characters in the leaves of *Syntrichia* species. We also measured hydration/dehydration curves to understand the rate of conduction and dehydration by experimental approaches. *Syntrichia* is an ectohydric moss that can externally transport and store water from the base of the stem using capillary action. We propose a new framework to study ectohydric capabilities, which incorporates three morphological scales and the timing of going from completely dehydrated to fully hydrated. Characters of interest in this model include cell anatomy (papillae development, hyaline basal cells and laminar cells), architecture of the stem (concavity and orientation) and whole clump characteristics (density of stems). We report significant variations in the speed of conduction, water holding capacity and hydration associated with each species studied (11 in total). All *Syntrichia* species are capable of external water conduction and storage, but the relevant traits differ among species. These results help to understand potential evolutionary and ecological trade-offs among speed of water conduction, water holding capacity, ontogeny, and differing habitat requirements. An integrative view of ectohydry in *Syntrichia* contributes to understanding the water relationships of mosses.

## Teaser

Terrestrialization imposed major challenges for photosynthetic organisms. Bryophytes adapted to move and store water externally (ectohydry) rather than through a specialized internal machinery (vascular system). This study provides the basis for understanding the main factors influencing ectohydry and understanding of species-specific morphological traits underlying functions of conduction or storage in the dryland moss *Syntrichia*. Better understanding of the roles of mosses and their water relations, in habitats where these plants form thick 'buffering' layers between the substrate and the atmosphere, will lead to better appreciation of their importance for water retention, storage and flow in dryland ecosystems

## Introduction

Terrestrialization imposed major challenges for photosynthetic organisms. The plesiomorphic condition for the earliest land plants, as judged by ancestral character state reconstruction, must have included a moderate form of desiccation tolerance (DT; Oliver *et al.* 2005; Mishler and Oliver 2009). *Desiccation tolerance* is the ability of the plant to revive after being air-dried at the cellular level (Alpert and Oliver 2002; Proctor *et al.* 2007; Koster *et al.* 2010). A related but distinct plesiomorphic condition is *poikilohydry*, the rapid equilibration of the plant’s water content to the surrounding environment (Kappen and Valladares 1999; Alpert and Oliver 2002). After they invaded land, embryophytes explored two contrasting paths for facing the dehydrating effects of the atmosphere and intermittent water supply (Proctor 2001; Nicklas 2016).

The first path, taken by several different lineages of bryophytes (i.e. liverworts, mosses and hornworts) was to retain poikilohydry while evolving new adaptations to tolerate free water loss, rather than prevent it, and to improve external water uptake (Proctor 1982, 2000). Most bryophytes tolerate desiccation by suspending their metabolism when dry and then rapidly resuming photosynthesis and growth during moist periods (Proctor 1979, 2001). The second path, taken by tracheophytes (vascular plants), was to evolve efficient internal machinery to move water from the soil to the canopy (referred to as *endohydry*; Proctor and Tuba 2002). Vascular plants evolved adaptations to prevent water loss (i.e. a cuticle and stomata closure) and conduct water internally via specialized internal tissues (xylem and phloem) from roots that absorb water and nutrients (Proctor 1982).

While a few moss lineages can be classified as endohydric, the vast majority of mosses are either ectohydric or mixohydric (Buch 1945; Proctor 1979). Endohydric species of mosses have well-developed water-conducting cells—hydroids—similar to the internal conducting systems of vascular plants (Hébant 1977; Ligrone *et al.* 2000). Water is absorbed from the substratum and conducted internally to the evaporative surface (Raven 2003); a pattern associated with a limited number of large moss genera (e.g. *Polytrichum* and *Dawsonia*; Tansley and Chick 1901; Bobdribb *et al.* 2020). Ectohydric mosses, on the other hand, rely on external water transport and absorption over the entire plant surface (Proctor 1979). Water is quickly absorbed and moves rapidly along the plant surface. This character allows mosses to take advantage of dew or fog by absorbing water directly from the atmosphere (Anderson and Bourdeau 1955; Glime 2017). A combination of a wettable surface with internal conduction is characteristic of mixohydric moss species (Buch1945; Proctor 1982). Even some of the large endohydric species also complement their internal water conduction with water that moves through capillary spaces found on the sheathing leaf bases (Bowen 1933; Mägdefrau 1935; Bayfield 1973; Proctor 2011).

External water conduction in ectohydric mosses requires pathways for water to move from one place to another, without completely covering all cell surfaces in water, which would not allow sufficient gas exchange (Proctor 1979, 2011). There are a variety of different ways that ectohydric mosses achieve water transport and storage. Overlapping leaves with sheathing bases can create a network of capillary spaces (Bowen 1931; Mägdefrau 1935; Buch 1947) and increase storage depending on the angle and concavity formed between the leaf and stem. Paraphyllia (small leaf-like structures scattered along the stem), or rhizoids (hair-like filaments on stems that anchor the plant to the substrate), can also increase the surface area and facilitate water transport (Proctor 2011). In some mosses, interspaces between papillae (tiny projections of the leaf surface) can reduce water tension and create a network of channels for an efficient capillarity movement across the leaf (Proctor1982; Proctor *et al.* 1998). Finally, the presence of hyaline leaf cells (dead and porose), found in several moss species (e.g. *Sphagnum* and *Leucobryum*), significantly increases water storage (Proctor 1979; Glime 2017).

Water uptake has been observed in mosses through observations and experimental procedures ranging from simply applying water to a clump of mosses (Anderson and Bordeau 1955; Sand-Jensen and Hammer 2012), to using dyes or radioactive tracers to track water movement in a particular tissue (Bowen 1931, 1933; Buch 1947; Sokolowska *et al.* 2017). Much attention has been centred on endohydric species due in part to their similarity to vascular plants. A few moss families, for example, Polytrichaceae and Mniaceae, have become model systems to study internal water relations in mosses (Pressel *et al.* 2006; Bobdribb *et al.* 2020; Duckett and Pressel 2020). However, mechanisms for water uptake, movement and storage need to be studied in ectohydric species as well, which are the great majority of moss species and represent a distinctive pathway to life on land as discussed above.

*Syntrichia* is a monophyletic group of mosses with more than 90 named species distributed in temperate regions worldwide ranging from deserts to alpine habitats (Mishler 1994; Gradstein *et al.* 2001; Jauregui-Lazo *et al.* 2022). This genus displays an extraordinary diversity of leaf characters, sizes and shapes (Zander 1993), which are partly related to water uptake and external conduction (Jauregui-Lazo *et al.* 2022). However, relationships between structure and function are complex. As *Syntrichia* acquires water, small chambers and capillary spaces fill up with water and the moss expands, making a completely new set of structural characteristics appear, ranging from modifications in the architecture of the whole plant to extensive changes in leaf stance and cell turgor. The capillary systems found together in *Syntrichia* suggest that some characters are interconnected. The way these characters function and/or reflect adaptation to different environmental regimes remains poorly understood.

This study aimed to (i) provide a new framework for understanding the dynamics of external water conduction using the genus *Syntrichia* as a model system, (ii) understand the dynamics of hydration and dehydration and (iii) discuss the implications of external water conduction for the overall ecology and life history trade-offs in mosses.

## Materials and Methods

### Plant material and pre-treatment

A total of 11 species of *Syntrichia* were studied; different species were selected for different experiments because of their suitability for evaluating particular aspects of water relations. These species, and the experiments they were used in, are given in Table 1. All stems were randomly sampled from recent herbarium specimens deposited at the University Herbarium at the University of California, Berkeley or Missouri Botanical Garden Herbarium as detailed in Table 1.

**Table 1.** Detailed description of the species used in this research, including species name, habitat preferences (type of substrate), conducted experiment (numbered according to the experiment section) and herbarium voucher (collector, collection number and herbarium).

Species	Habitat preferences	Conducted experiment	Herbarium voucher
*S. amphidiacea* (Müll. Hall.) R. H. Zander	Commonly found in wood and rocks	Exp. #4	Brinda, J. C., 8680, UC.
*S. bartramii* (Steere) R. H. Zander	Commonly found in rocks	Exp. #1, 2, 4	Brinda, J. C., 8182, UC.
*S. calcicola* J. J. Amann	Commonly found in rocks	Exp. #3, 4, 5	Norris, D. H., 104694, UC
*S. campestris* (Dusén) R. H. Zander	Only in soil	Exp. #3, 4	Jauregui-Lazo, J., 471, UC
*S. caninervis* Mitt.	Only in soil	Exp. #3, 4	Brinda, J. C., 9434, UC
*S. chisosa* (Magill, Delgad and L. R. Stark) R. H. Zander	Commonly found in wood and rocks	Exp. #4	Brinda, J. C., 2539a, MO
*S. papillosa* (Wilson ex Spruce) Spruce	Commonly found in wood	Exp. #4	Brinda, J. C., 7142, UC
*S. papillosissima* (Copp.) Loeske	Commonly found in soil	Exp. #1, 2, 3, 4	Brinda, J. C., 2209, UC
*S. princeps* (De Not.) Mitt.	Commonly found in soil	Exp. #1, 2	Brinda, J. C., 8400, UC
*S. ruralis* (Hedw.) F. Weber and D. Mohr	Commonly found in soil	Exp. #3	Brinda, J. C., 9108, MO
*S. squarripila* (Thér.) Herzog ex Brinda, Jáuregui-Lazo and Mishler	Only in soil	Exp. #3	Larrain, J., 42093, MO

Before all experiments, the mosses were hydrated in the laboratory at 12 °C in petri dishes for a week under a photoperiod of 12 h day and 12 h night with light intensity of 100 PAR. The gametophytes were sprayed three or four times a week with deionized water until further use to reactivate metabolism, and were slowly dehydrated (15 °C at 60 % HR for 24–48 h) before microscopic examinations and/or further experiments. All samples entered the experiments in the same state of hydration and health.

### Experiments conducted

#### Fine-scale anatomical structures.

 We sampled completely developed leaves from the upper section of the stem for compound, scanning electron and confocal microscopic observations (five stems per species). Samples were hand-sectioned with a razor blade for a cross-section of the leaves. We created permanent slides for future use and comparison. We used a Quanta 3D FEG 200/600 machine (from FEI Company, USA) for both scanning electron microscope (SEM) and environmental scanning electron microscope (ESEM) microscopic observations. The set-up conditions are detailed in **Supporting Information—Table S1**.

#### Tracers.

We sampled dehydrated and well-developed leaves and stems from gametophyte tissue and applied two tracers to observe water uptake, movement and storage. First, we used methylene blue to increase contrast and visualize the movement of the water when applied to different parts of the moss. We performed the visualization of the perforated hyaline basal cells after 10 min of submerging the moss tissue in a solution of methylene blue.

Second, we exposed leaves to 1 % lucifer yellow (LYCH; ThermoFisher, USA) to detect apoplastic transport as described in Bederska *et al.* (2012). After an exposure of 10–15 min with LYCH, the tissue was washed with distilled water. The whole leaf and cross-sections were mounted in water and analysed with confocal microscopy. We used a Zeiss LSM 710 (Carl Zeiss Inc., USA) laser scanning confocal microscope using 10×, 20× and 40× objectives. The emission spectrum was between 535 and 575 nm for LYCH. The images from confocal microscopy were processed by Imaris 3-D software (Oxford Instruments, UK) at the Biological Imaging Facility at the University of California, Berkeley.

#### Hydration curves.

 We used a transparent 96-well plate to apply a predetermined amount of water (1, 3, 8, 15, 30, 70 and 110 µL) to the base of a shoot in order to observe the overall water uptake and upward movement towards the tip of the stem. The stems were at least 5 mm in height, clean, green and fully developed. We carefully placed each stem in each well and recorded water movement. We measured the initial and final weight (mg), height (mm) and the vertical distance travelled by water along the stem (mm). We calculated the water content ([{final weight (wet) − initial weight (dry)}/initial weight (dry)] × 100) in relation to the dry weight (DW) at a given amount of water as suggested by Anderson and Bourdeau (1955).

#### Speed of conduction and water holding capacity.

The speed of conduction was calculated by measuring the rate of external water conduction to cover the full height of the stem (from the bottom to the tip) until the moss shoot reached maximum storage capacity (when the stem and leaves are fully expanded and stop absorbing and distributing water; eight stems per species). We applied 15 µL of water to measure the speed of conduction. Then, the WHC at full turgor was measured as water content (mean of maximum water content, % DW) at this point as described in the previous section.

#### Dehydration curves.

 The effect of the density of stems on drying rate was measured by placing individual stems of *Syntrichia calcicola* of similar weight and size into a simulated clump. We used a woven stainless-steel mesh of 10 mm^2^ with 1 mm^2^ holes to place individual stems. We used three densities with four replicates each. Density one (Clump 1) consisted of 40–50 stems per 10 mm^2^ in which all stems touched each other when hydrated (simulating a dense clump); Density two (Clump 2) consisted of 20–25 stems scattered around with minimal proximity when hydrated, and Density three (Clump 3) consisted of 10 stems per 10 mm^2^ scattered with no stems touching when fully hydrated. The clumps were hydrated from their base and placed for slow dehydration in a controlled room (at 12 °C and 40–60 % under a photoperiod of 12 h day and 12 h night, and 100 PAR).

### Statistical analysis

To compare species in Experiment 4 for speed of conduction (mm s^−1^) and water-holding capacity (WHC; % DW), we used a non-parametric Kruskal–Wallis test. We used four replicate stems for each species. Subsequently, we performed individual comparisons (two independent groups) of the means for the two metrics using the Wilcoxon test in R 4.1.0. To plot the speed of conduction and WHC against the proportion of basal cells, we used a linear regression model in R 4.2.2 and Type I ANOVA to test the significance of the variables **[see****Supporting Information—Figure S1**].

For Experiment 3, we used a non-linear regression model in R 4.1.0 to plot hydration curves comparing water content (in % DW) to each amount of water added. For Experiment 5, we used a non-linear regression model in R 4.1.0 to plot dehydration curves comparing water content (in % holding capacity) to dehydration time.

## Results

### Fine-scale anatomical structures

**Figure 1.**
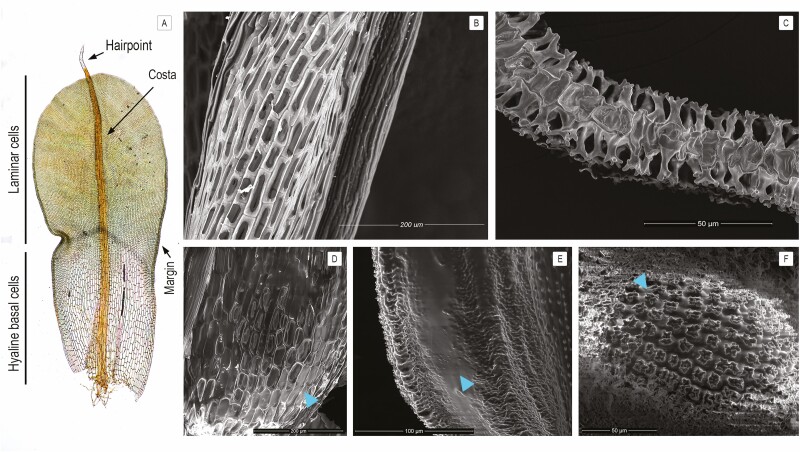
(A) Leaf of *S. princeps* indicating the differentiation between laminar and hyaline basal cells, as well as the hairpoint, costa and margin. (B, C) Scanning electron microscope images of dry *S. papillosissima* show the elongated and perforated basal cells (B) and papillae in a cross-section (C). (D–F) Environmental scanning electron microscope images of wetting *S. princeps* showing the initial stage of hydration in the hyaline basal cells (D), laminar cells (E) and interspaces among papillae (F). Light blue triangles indicate the capillary spaces in contact with the free water.

The leaves of *Syntrichia* are composed of two main types of cells; photosynthetic upper laminar cells and large, hyaline basal cells (Fig. 1A). The laminar cell size varies from 5 to 15 µm. The hyaline basal cells are quite distinct from the laminar cells; they are clear with elongated thin-walled cells and frequently perforated upper and lower surfaces (Fig. 1B). Both sides of the laminar cells have bulging surfaces to a greater or lesser extent and also bear papillae, which are hollow, usually branching, projections of the cell. Together, these cell projections create intercapillary spaces on the leaf surface from 3 to 10 µm wide (Fig. 1C). The ornamentation of laminar cells is species-specific. *Syntrichia bartramii* has a relatively flat cell surface and shorter papillae as compared to *S. princeps* or *S. papillosissima*. The cell surface of *S. princeps* is bulging and has numerous branched papillae per cell. In contrast, *S. papillosissima* has an extremely bulging cell surface but only a single large papilla with two successive branches (Fig. 1C).

The initial contact of water with hyaline basal cells and papillae is shown in Fig. 1D–F for *S. princeps* (similar observations were made for *S. papillosissima* and *S. bartramii*). The images derived from ESEM suggested that water filled up the hollow spaces of the hyaline basal cells as indicated with light blue arrows in Fig. 1D. The small spaces among papillae formed a network of capillary spaces where the water began to fill and become distributed along the leaf lamina. The recurved portion of the leaf margin was an additional location where the water began to move rapidly and distribute up the leaf.

### Tracers

Leaves of *S. papillosissima*, *S. princeps* and *S. bartramii* were able to absorb and transport the methylene blue and LYCH. Methylene blue was thus an efficient tracer for observing external water movement in the moss shoot. When water containing the dye was applied to the base of the shoot, the water moved upward rapidly. The water first moved externally up to the tip of the stem while moving through and filling up the hyaline basal cells and the spaces among the sheathing leaf bases, rhizoids and the stem. Then the hydration process began to reach the leaf laminae as the water moved upward on the leaves via the capillary channels described above. In the dry state, the folded lamina and recurved margin of the leaf initially absorbed water faster than other locations of the lamina. As the leaf opened to its wet state, water movement then occurred more generally across the lamina until reaching maximum capacity. **Supporting Information—Video 1** shows the hydration of an individual stem in S. *papillosissima*.

Lucifer yellow proved useful to trace hydration in the walls of laminar cells. At full hydration, LYCH was located in the leaf lamina, specifically in the apices of the papillae, and covered the whole hairpoint. LYCH was slightly bound to other parts of the cell, such as the area between the papillae, and associated with the wall of the hyaline basal cells. The processes of external water movement using methylene blue dye and 3D reconstructions of the reaction between LYCH and the different anatomical characters are documented in **Supporting Information—Videos 2 and 3**.

### Hydration curves

**Figure 2.**
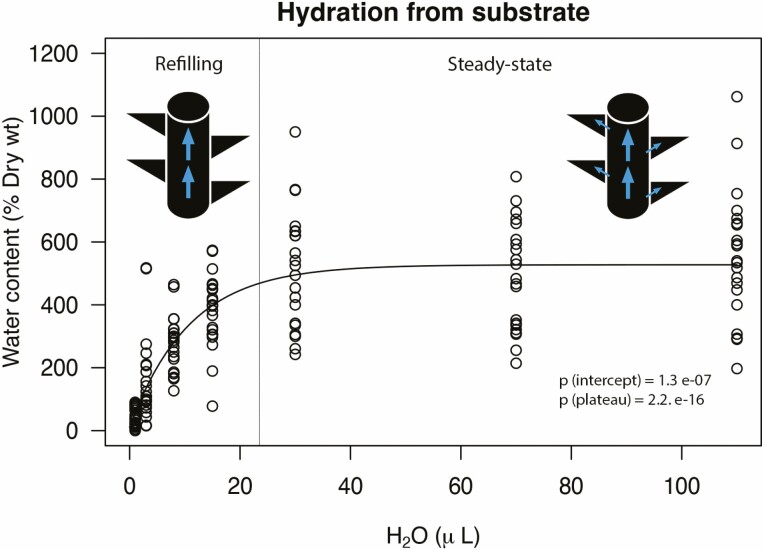
Water content of stems of six *Syntrichia* species (*S. campestris*, *S. calcicola*, *S. caninervis*, *S. papillosissima*, *S. ruralis* and *S. squarripila*) depending on the amount of water applied to the base of the moss shoot.

An asymptotic non-linear regression curve of hydration of the moss shoots for six *Syntrichia* species (*S. campestris*, *S. calcicola*, *S. caninervis, S. papillosissima*, *S. ruralis* and *S. squarripila*) is shown in Fig. 2. There appeared to be two distinct phases: a rapid increase (refilling) followed by a more gradual approach to a steady state of water content. In the initial phase, there was a rapid increase in water content from complete dehydration. The moss shoots rapidly surpassed more than 100 % of its DW with only a very small amount of water (8 µL). This initial phase was characterized by vertical water conduction along the shoot from the bottom to the tip, followed by lateral capillary movement along the leaves as described in the previous section. The second phase began at the inflection point in the curve, where water content was around 30 µL. This phase appeared to be reaching a steady state that is characterized by achieving the maximum capacity for water content and a homogeneous distribution of water along the stem and leaves. Hydration culminated with no additional absorption at maximum WHC (300–1000 % of DW).

### Speed of conduction and WHC

**Figure 3.**
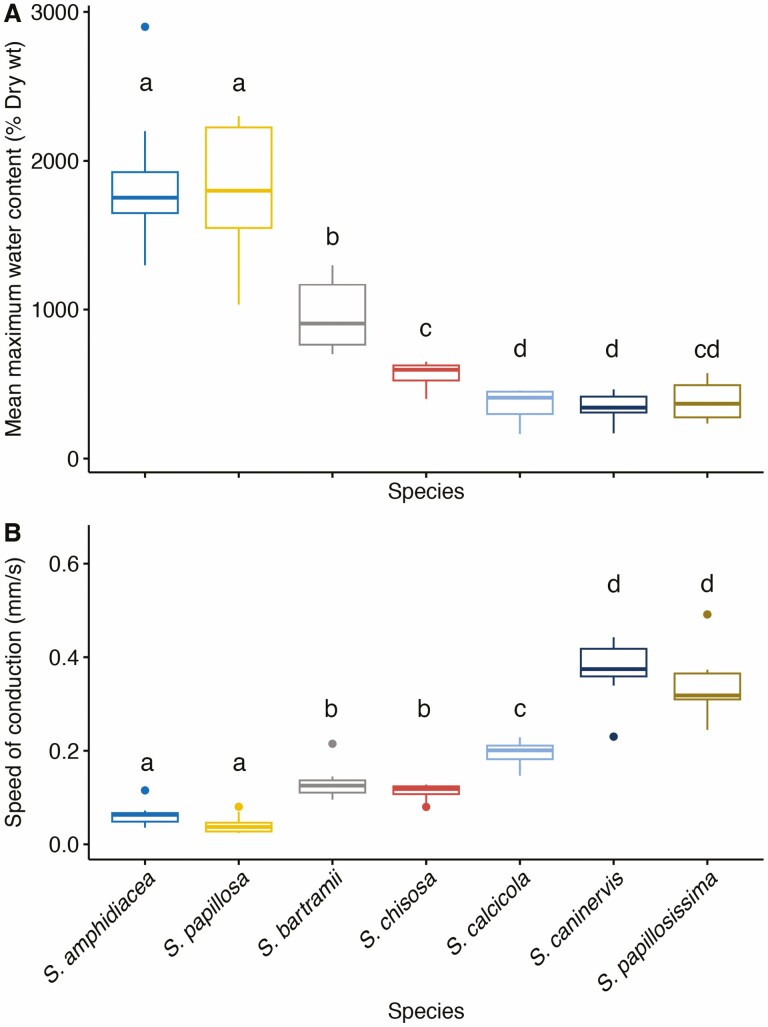
Water holding capacity (mean maximum water content, % DW) (A) and speed of external water conduction (mm s^−1^) (B) compared among different species of *Syntrichia* using a non-parametric Kruskal–Wallis test. Different lowercase letters above box plots indicate a significant pairwise comparison (Wilcoxon test, *P* < 0.05), while the same letters indicate a non-significant pairwise comparison.

The WHC and speed of external conduction differed significantly among species of *Syntrichia* (Kruskal–Wallis test, *P* = 1.7e^−8^ and *P* = 4.1e^−9^, respectively; Fig. 3). The WHC showed an opposite trend to the speed of external conduction. For instance, *S. amphidiacea* (1867 % DW) and *S. papillosa* (1804 % DW) had significantly higher water contents at full turgor (Fig. 3A) than other species, but the lowest speed of external water conduction (0.06 and 0.0.4 mm s^−1^, respectively) (Fig. 3B). In contrast, *S. papillosissima* (391 % DW), *S. calcicola* (366.8 % DW) and *S. caninervis* (349 % DW) showed significantly low WHC compared to the rest of species, but high speeds of conduction. *S. caninervis* (0.37 mm s^−1^) and S*. papillosissima* (0.34 mm s^−1^) had significantly higher speeds of external transport (Fig. 3B). *Syntrichia bartramii* and *S. chisosa* had intermediate mean values of WHC (956 and 565.1 % DW, respectively), and speed of external conduction (0.13 and 0.11 mm s^−1^, respectively). However, they only differed significantly in their water content at full turgor (*P* = 0.001).

### Dehydration curves

**Figure 4.**
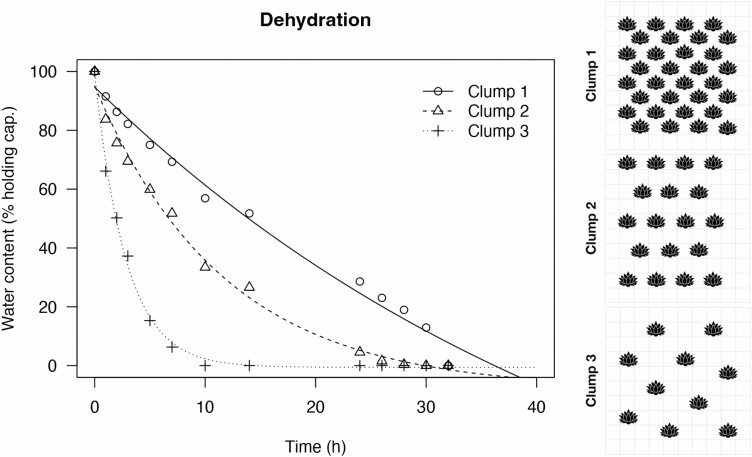
Dehydration curve of three clumps of *S. calcicola* with varying densities of shoots. Clump 1 is a dense clump, similar to a clump growing in nature; Clump 2 is a modified clump with ~50 % of the shoots of Clump 1; Clump 3 is a less dense clump with 25 % of shoots of Clump 1.

To visualize the decrease in water content over time as clumps dried, we fitted curves using an asymptotic non-linear regression model. The three clumps differed in rate of dehydration as shown in Fig. 4. The densest clump (Clump 1) experienced a gradual decrease in water content, while the less dense clumps had a drastic decrease in water content. The denser clumps (Clumps 1 and 2) held water that remained significantly longer than in the clump that consisted of scattered stems (Clump 3; Fig. 4, at the right). The least dense clump (Clump 3) was almost completely dried after 10 h while the other clumps did not dry till after 25 h.

## Discussion

### Structure and function of the external capillary system in *Syntrichia*

Traits related to the external water conduction process vary among the species of *Syntrichia* studied here. The hyaline basal cells, ornamentation of papillae and the hairpoint represent crucial anatomical characteristics that influence water relations at a fine scale. Microscopic observations with a SEM of leaves of *Syntrichia* showed that the hyaline basal cells are dead, hollow and in some cases have perforated surfaces (Fig. 1). We suggest that these cells function in the refilling of water throughout the stem, providing a vertical pathway for fast uptake in phase 1 of the hydration curve (Fig. 2). These cells varied strongly across the species studied. *Syntrichia bartramii* has a low proportion of hyaline basal cells compared to the large, elongated and extended proportion of the hyaline basal cells observed in *S. papillosissima* and *S. princeps*. Jauregui-Lazo *et al.* (2022) examined how these hyaline basal cells evolved across the phylogeny of *Syntrichia*, and how this variation was potentially associated with different strategies for absorbing and conducting water.

The mammillae and papillae of *Syntrichia* are distinct characters, often varying independently (Mishler 1987; Kou *et al.* 2014), and forming capillary spaces (Proctor 1982; Proctor *et al.* 1998). Here, we demonstrated, using ESEM images, that this network of capillary spaces can canalize water movement across the leaf lamina, corroborating previous observations in *S. ruralis* (Proctor 1982; Pressel and Duckett 2011) and *S. intermedia* (Dilks and Proctor 1979). The microscopic observations of the hairpoint suggested that they can be smooth or spinulose. The hairpoint captures small drops of water from the atmosphere **[see**Supporting Information—Video 4) as first pointed out by Pan *et al.* (2016). The grooves from the projections in a spinulose hairpoint are able to form nanodrops of free water. As the drops of water expand, they can be conducted towards the lamina and become available to the moss (Pan *et al.* 2016). Moreover, Tao and Zhang (2012) showed that the presence of the hairpoint influenced water relations in the moss *Syntrichia* by increasing water content and delaying evaporation rates.

**Figure 5.**
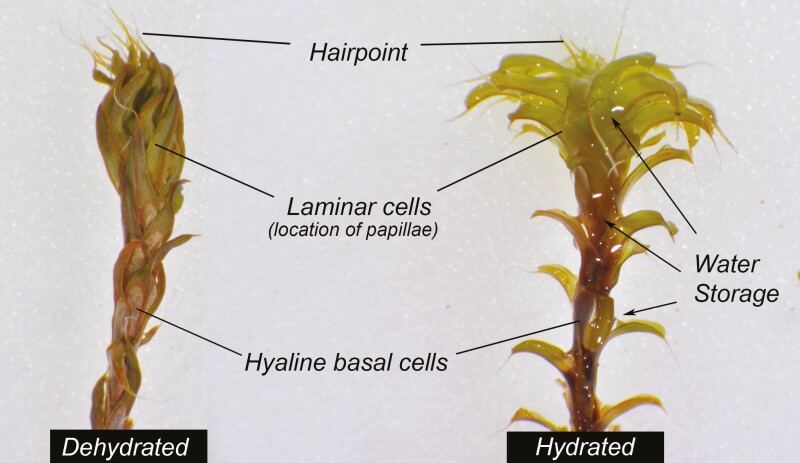
Two extremes of a morphological hydration spectrum in the moss *S. papillosissima*. Note the morphological differences between a fully dehydrated and hydrated stem and water storage capacity.

The individual stem has a wide range of morphologies depending on the level of hydration. There is a striking difference in *Syntrichia* leaf arrangement when dehydrated or hydrated (Fig. 5). When dry, leaf orientation can be crisped, twisted, keeled or imbricated and may affect initial water uptake. For example, the combination of being keeled and twisted increases the total amount of space available to distribute water along the ventral surface of the lamina. Whereas once the leaf is hydrated, the new leaf angle and shape may increase WHC (Wu *et al.* 2014). For example, *S. caninervis* spreads its leaves rapidly from an imbricate to a squarrose arrangement after rewetting, increasing water storage and light capture (Wu *et al.* 2014). Presumably, the change in volume is mostly in the upper leaf lamina since those cells are able to shrink completely when dry (Proctor *et al.* 1998) while hyaline cell shape remains intact. Because hyaline basal cells sheathe the stem, and are perforated, they can provide an efficient external conducting system up the stem. A large proportion of hyaline basal cells making up the leaf is associated with higher speed of conduction along the stem **[see****Supporting Information—Figure S1**. The individual stem can act as a columnar pipe armed with the overlapping sheathing bases of leaves for water to be conducted efficiently. **Supporting Information—Videos 5 and 6** show the stages of hydration and dehydration of several stems of *S. calcicola* and *S. caninervis*, respectively.

The organization of *Syntrichia* clumps in nature is of variable density, and while all species in *Syntrichia* can hold and transport water externally to some extent, a hydration event is influenced by the density of a clump. This study showed that high densities of stems hold water longer than low densities. In addition to the fine capillary system in each stem, water is shared by adjacent stems within the clump forming an additional level in the capillary system.

**Figure 6.**
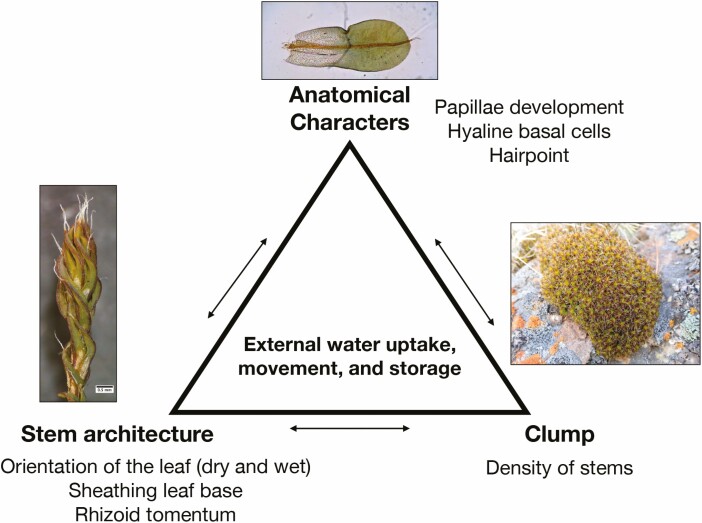
An integrated model for external water uptake, movement and storage using as an example the dryland moss *Syntrichia*. Leaf anatomical characters (e.g. papillae), stem architecture (e.g. sheathing bases) and clump structure are three interconnected functional aspects of mosses that influence water relations.

Based on these results, we propose a new integrated framework for understanding the link between morphology and water relationships in *Syntrichia*, which includes three morphological scales (Fig. 6). At the finest scale, leaf anatomy has three main characteristics that can be related to water uptake, conduction and storage. At a larger scale, the individual stem possesses both hydrated and dehydrated architecture that influences water relations. Finally, the proximity of stems largely influences the clump’s water capabilities (e.g. holding water capacity) in a given environment.

### Habitat preferences in relation to ectohydry in *Syntrichia*

Variations in morphological and anatomical characteristics appear to be at least partly associated with habitat preferences in *Syntrichia*. Epiphytic species in *Syntrichia* vary in morphology compared to terricolous counterparts (Jauregui-Lazo *et al.* 2022). On average, epiphytic species studied here (*S. amphidiacea* and *S. papillosa*) held almost two times as much water as saxicolous species (*S. chisosa* and *S. bartramii*) and more than four times more than terricolous species (*S. calcicola*, *S. caninervis* and *S. papillosissima*). This extraordinary holding capacity has been previously reported in *Tortula intermedia* (*S. intermedia*) (Dilks and Proctor 1979) and *T. ruralis* (*S. ruralis*; Proctor *et al.* 1998). On the other hand, the terricolous species had a higher speed of external water transport than either epiphytic or saxicolous species. *Syntrichia caninervis* had a significantly higher speed of external transport than other soil-dwelling species, such as *S. papillosissima* and *S. calcicola*. However, there was no difference either between *S. papillosa* and *S. amphidiacea* (epiphytic species) or between *S. chisosa* and *S. bartramii* (saxicolous species) species since they have very similar responses to external conduction.

There may be a trade-off between speed of conduction and WHC at full turgor, potentially associated with habitat preferences. Zotz (2016) suggested that a conglomerate of traits (rather than a single one) makes a plant epiphytic, including bryophytes inhabiting tree substrates (Fedosov *et al.* 2020). Plants of *Syntrichia* inhabiting trees are commonly bulbiform, have a short hairpoint (or none) and have a reduced proportion of basal hyaline cells that may or may not be perforated. But some species such as *S. amphidiacea* and S. *bartramii* inhabit both rocks and trees, and demonstrate no clear pattern regarding habitat preferences and water relations. Future studies comprising a larger set of species need to be conducted, in a phylogenetic context, to address potential adaptive relationships between habitats, water relations and plant morphology.

The ontogeny of the moss gametophyte might also contribute to the explanation of different responses to hydration and dehydration in relation to morphology. A distinct heteroblastic series occurs as a stem matures in *Syntrichia* (Mishler and Luna 1991). Different types of leaves are produced depending on the ontogenic stage of the shoot. For instance, the base of the shoot is characterized by juvenile leaves that have no clear distinction between basal and laminar cells, and no hairpoint, while the upper part of the shoot consists of mature leaves with a clear distinction between basal and laminar cells, and a hairpoint (Mishler 1986). As an acrocarpous moss, new shoots develop just below where the original shoot forms archegonia/antheridia at its tip. Thus, the heteroblastic series culminates in archegonia or antheridia. Therefore, if a species relies on asexual reproduction instead of sexual reproduction, as many epiphytic *Syntrichia* do, they may evolve to become neotenic and stop their development at a juvenile stage (Mishler 1986). Many epiphytes have short, condensed stems bearing a juvenile form of leaves (Fedosov *et al.* 2020), i.e. short or no hairpoint, flat leaf lamina and a short proportion of hyaline basal cells (Mishler 1986; Jauregui *et al.* 2022). These morphological traits do not capture, absorb or conduct water efficiently, but retain water well. Further studies need to address potential evolutionary trade-offs caused by the heteroblastic development between asexual reproduction and juvenile morphology compared to sexual reproduction and mature morphology.

### Comparisons with external conducting systems in other mosses

Capillary conducting systems are diverse across mosses. The development of the papillae could be characterized by the number of projections per cell, the presence of lumen and ornamentation (i.e. branching type; Câmara and Kellog 2010; Glime 2017). Examples of papillae are found in many unrelated groups of acrocarps, such as *Encalypta* (Encalyptaceae)*, Pleurochaete, Syntrichia, Triquetrella* (Pottiaceae) and *Orthotrichum* (Orthotrichaceae; Proctor 1979; Castaldo-Cobianchi and Giordano 2013; Kou *et al.* 2014), and pleurocarps (e.g. Thuidiaceae, Theliaceae, Leskeaceae and Sematophyllaceae; Hedenäs 2001; Câmara and Kellog 2010). Despite the diversity of organization of papillae, most create an interconnected network of channels ranging from 2 to 10 µm wide (Proctor 1979, 1982; Pressel and Duckett 2011), which are able to sustain an extraordinary long-distance water transport (Proctor 2011).

The diffusion of CO_2_ is about 10 000 times slower in water than in air, thus external water conduction creates a potential problem for bryophytes if their surfaces are completely covered in water (Dilks and Proctor 1979). Indeed, net photosynthesis showed a decrease at extraordinary levels of water content (1000–1500 % of DW; Dilks and Proctor 1979; Green *et al.* 2011). Papillae may thus also function in gas exchange, by allowing higher CO_2_ exchange when water is distributed across the lamina (Proctor 1979). Figure 2 shows how the water is distributed along the leaf surface in *Syntrichia* while some apices of the papillae remain uncovered (Dilks and Proctor 1979). **Supporting Information—Videos 2 and 3** show how lucifer yellow dye reacts with the apices of the papillae in *Syntrichia* because those areas could be hydrophobic in the rewetting phase. Proctor (1979) suggested that plants with squarrose or spreading leaves (and a concave shape) have a water-repellent lower surface and/or papillae for efficient gas exchange while the upper surface remains saturated (Green *et al.* 2011). Future research needs to characterize the epidermal surface of the papillae from the base to the tip to know whether differentiation of the cuticle or wax thickness exists.

The most well-known example of the capacity of mosses for holding and releasing water slowly comes from *Sphagnum* (Bengtsson *et al.* 2020). This moss has two types of leaf cells (chlorophyllous and hyaline cells), which contribute to an unusual system for water storage and normal growth (Glime 2017). There are some similarities in the form of the hyaline perforated cells between *Syntrichia* and *Sphagnum* (thin-walled cell with perforations), however, their functions might be distinct. Since the hyaline basal cells of *Syntrichia* exist in a separate part of the leaf (sheathing the stem) and are perforated, they can provide an efficient external conducting system up the stem, acting as a vertical pathway for fast uptake, rather than functioning in water storing as seen in *Sphagnum* (Hájek and Beckett 2008; Bengtsson *et al.* 2020).

One of the most remarkable traits of some mosses that has received much attention is the hairpoint. The hairpoint might function in several ways. Pan *et al.* (2016) demonstrated that hairpoint is able to capture small nanodrops of water from the atmosphere and conduct them towards the lamina in *S. caninervis* and suggested this may be an adaptation in mosses living in xerophytic environments. In addition, the hairpoint may function to minimize water loss by reflecting the sunlight and creating a boundary layer (Scott 1982; Tao and Zhang 2012). Conversely, the hairpoint may function to repel water when the amount of water is not sufficient to wet the plants fully, and thus, they would be damaged by breaking dormancy without being able to recover. While we didn’t analyse the hairpoint in an experiment to test such hypotheses, this structure has peculiar water affinities, as seen in **Supporting Information—Video 4**, that is likely related to how the cell wall is formed and organized.

Leaf arrangement along the stem is also an important character of plant architecture that influences both photosynthesis and water use (van Zanten *et al.* 2010). Buch (1945) suggested that the spaces among leaves, such as the spaces among overlapping sheathing bases and the stem, can form a type of macrocapillary system. This storage capacity of the junction between stem and leaf can be seen in *Syntrichia* and its relatives (Fig. 5). In addition to the leaf arrangement, the rhizoidal tomentum on the stem may also provide capillary spaces to store and conduct water towards the tip of the stem in many mosses, such as Dicranales and Bryales (Pressel and Duckett 2011). Differences in stem anatomy between *Pleurozium schreberi* and *Hylocomium splendens* revealed a distinct pathway for internal water conduction via apoplastic and symplastic transport using fluorescent tracers (Sokolowska *et al.* 2017). We would emphasize the importance of the leaf arrangement in both a dry and wet stage because they each influence water relations at different times, as we show in Fig. 5.

Clump structure (growth form) is noticeably different across various mosses and causes differing interactions with water (Sand-Jensen and Hammer 2012). Clump structure is quite important for water absorption and retention (Rice and Schneider 2004). For example, *Grimmia pulvinata* grows as a cushion in different sizes. A larger cushion has a thicker boundary layer and a lower surface-to-volume ratio that confers lower evaporation rates (Zotz *et al.* 2000), an explanation that is consistent with what we showed in *Syntrichia*. Clump structure influences air flow near the boundary layer; thus, the roughness of the canopy is critical to increasing or decreasing evaporation rates (water flux; Jenkins and Proctor 1985; Rice *et al.* 2001). In general, greater roughness of a cushion tends to create turbulent air flow near the moss reducing the boundary layer and increasing evaporation rates (Rice and Schneider 2004), leading to a greater dehydration rate in a clump. In natural conditions, the density of the clump as a whole determined the drying rate in a study of subarctic bryophytes (Elumeeva *et al.* 2011). Mosses also tend to grow compactly in dry habitats, like *Syntrichia*, thus reducing drying rates and holding water by adjacent shoots within the canopy to form an integrated capillary network (Nakatsubo 1994; Rice and Schneider 2004; Rice 2012). We infer that the effect of individual shoot morphology on water properties may often be marginal as compared to the clump structure.

### Future work is needed on the relationship of ectohydry to ecology and evolution

Ectohydry is a complex phenomenon where multiple factors of morphology play a role in space and time. In a recent survey, Patiño *et al.* (2022) highlighted that the function of morphological features, such as hairpoints, paraphyllia and paraphyses, in relation to fitness and physiological performance remains open as one of 50 fundamental questions in bryology. Here, we suggest a conceptual model to analyse the external water relationships of mosses in an integrated manner (Fig. 6). Future studies are needed to survey traits relating to external water conduction more widely among mosses. In addition, the evolution of environmental preferences needs to be studied in more detail. Once this additional information is available, phylogenetic comparative methods should be applied to determine the evolutionary origins of structural traits in relation to the environment present at that time. In this way, truly adaptive changes in evolution can be discovered.

## Supporting Information

The following additional information is available in the online version of this article—

**Table S1.** Optimal setting for the scanning electron microscopy (SEM) and environmental scanning electron microscope (ESEM) based on *Syntrichia* specimens.

**Figure S1.** Relationship between basal cells (explanatory variable) and speed of conduction (mm/s) (A) and mean maximum water content (% dry weight) (B). Linear regressions were tested with ANOVA test. Type I ANOVA on both models returned significant *p*-values for proportion of basal cells vs speed of conduction (*p* = 1.14e^−8^) and for proportion of basal cells vs mean of maximum water content (*p* = 3.69e^−7^). Shaded area represents 95 % CI.

plad025_suppl_Supplementary_Figure_S1

plad025_suppl_Supplementary_Table_S1

## Data Availability

The datasheets and codes for data analyses, and Supporting Information are available at Dryad: https://datadryad.org/stash/share/kMcfezUpXvd4tqBus-Aodh1TAXEmSrX8QjT9YLwD9WI. Datasheets used for the analyses mentioned in experiments conducted in sections ‘Hydration curves’, ‘Speed of conduction and water holding capacity’ and ‘Dehydration curves’ (Materials and Methods) are Hydration_curve.csv, WHC&Speed.csv, dehydraton_curve.csv and Correlation_traits&cell.csv. The script using previous datasheets is Script_22159S1.R that provide detailed explanation for statistical analyses and creation of figures. **Supporting Information—Video 1** (Suppl_V1): External water movement along an individual stem shows some important morphological characters for external water movement and storage (the hyaline basal cells, keeled leaves, papillae and recurvature of leaf margin) when free water is applied from the base in *S. papillosissima*. **Supporting Information—Video 2** (Suppl_V2): 3-D reconstruction of papillae in *S. bartramii* using LYCH as a dye in confocal microscopy. **Supporting Information—Video 3** (Suppl_V3): 3-D reconstruction of papillae in *S. papillosissima* using LYCH as a dye in confocal microscopy. **Supporting Information—Video 4** (Suppl_V4): Hydration of the clump when spraying small drops of water in *S. calcicola*. **Supporting Information—Video 5** (Suppl_V5): Hydration and dehydration of a clump when free water is applied from the base in *S. calcicola*. **Supporting Information—Video 6** (Suppl_V6): Hydration and dehydration of a clump when free water is applied from the base in *S. caninervis*.

## Notes

**Form and Function. Chief Editor:** Kate McCulloh
