# Supplementary figures and images for: The dynamics of external water conduction in the dryland moss Syntrichia
Source: AoB Plants. 2023 May 22;15(3):plad025. doi: 10.1093/aobpla/plad025 (PMC10244898; doi:10.1093/aobpla/plad025)

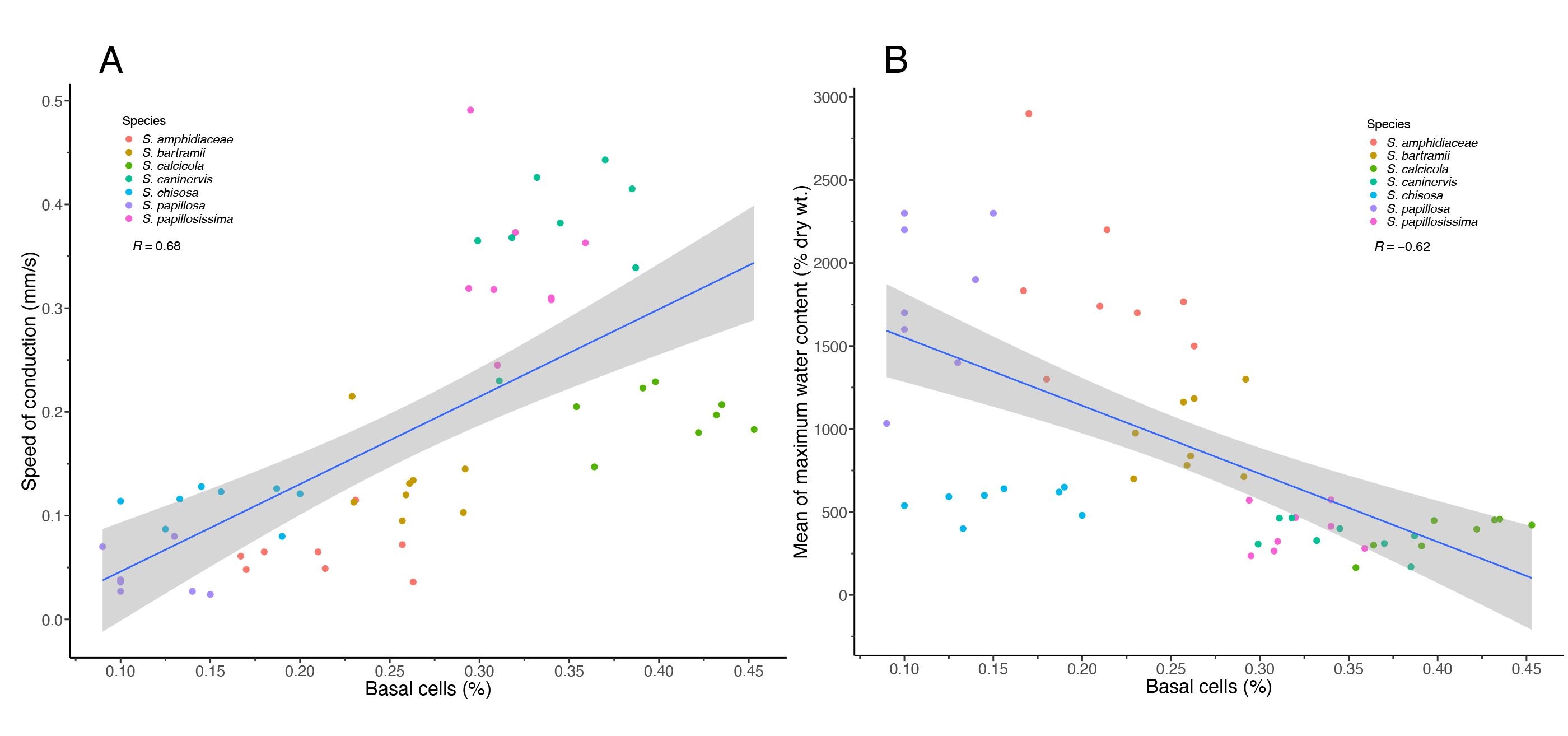

Supplement: plad025_suppl_Supplementary_Figure_S1 [file plad025_suppl_supplementary_figure_s1.jpeg]
