# Supplementary material for: The dynamics of external water conduction in the dryland moss Syntrichia
Source: AoB Plants. 2023 May 22;15(3):plad025. doi: 10.1093/aobpla/plad025 (PMC10244898; doi:10.1093/aobpla/plad025)
Supplement: plad025_suppl_Supplementary_Table_S1 [file plad025_suppl_supplementary_table_s1.docx]

| Vacuum mode | Beam settings (adjustments) | Notes |
| --- | --- | --- |
| HiVac (SEM) | Detector: ETD  Accelerating voltage: 5-10 [kV]  Spot size: 3.5-4.5  Aperture size: 30 [µm]  Beam current: 0.01 [nA]  Pressure: <10 Pa | The specimen for High Vacuum mode must be able to withstand a low-pressure environment. It must be clean and conductive. We used a coated sample with Argon. |
| ESEM | Detector: GSED  Accelerating voltage: 10-15 [kV]  Spot size: 5 - 6  Aperture size: 30 [µm]  Beam current: 0.1 [nA]  Pressure: ~600 Pa | All the specimens were in fresh state. |

Table S1. Optimal setting for the scanning electron microscopy (SEM) and environmental scanning electron microscope (ESEM) based on *Syntrichia* specimens.
